# 3-Ethyl­sulfinyl-2-(3-fluoro­phen­yl)-5,6-methyl­enedi­oxy-1-benzofuran

**DOI:** 10.1107/S1600536812044832

**Published:** 2012-11-14

**Authors:** Hong Dae Choi, Pil Ja Seo, Uk Lee

**Affiliations:** a Department of Chemistry, Dongeui University, San 24 Kaya-dong, Busanjin-gu, Busan 614-714, Republic of Korea; b Department of Chemistry, Pukyong National University, 599-1 Daeyeon 3-dong, Nam-gu, Busan 608-737, Republic of Korea

## Abstract

In the title compound, C_17_H_13_FO_4_S, the 3-fluoro­phenyl ring makes a dihedral angle of 6.14 (5)° with the mean plane [r.m.s. deviation = 0.008 (1) Å] of the benzofuran fragment. In the crystal, mol­ecules are linked by weak C—H⋯F, C—H⋯O and C—H⋯π inter­actions, forming a three--dimensional network. The crystal structure also exhibits slipped π–π inter­actions between the 3-fluoro­phenyl rings of neighbouring mol­ecules [centroid–centroid distance = 3.769 (2) Å and slippage = 1.684 (2) Å].

## Related literature

For background information on and the crystal structures of related benzofuran compounds, see: Choi *et al.* (2010 ▶); Seo *et al.* (2011 ▶).
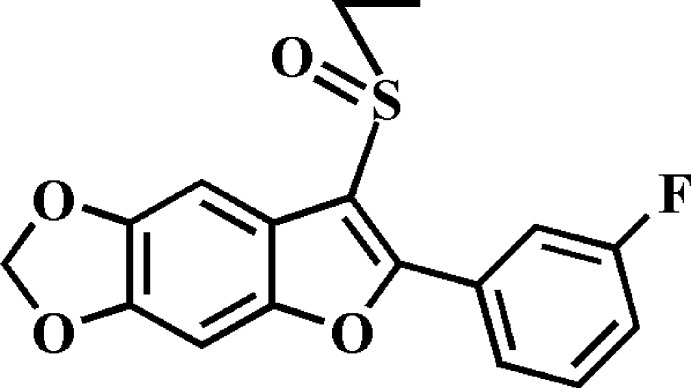


## Experimental

### 

#### Crystal data

C_17_H_13_FO_4_S
*M* *_r_* = 332.33
Monoclinic, 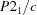

*a* = 8.8516 (2) Å
*b* = 21.8221 (4) Å
*c* = 7.7228 (2) Å
β = 102.949 (1)°
*V* = 1453.80 (6) Å^3^
*Z* = 4
Mo *K*α radiation
μ = 0.25 mm^−1^
*T* = 173 K
0.39 × 0.29 × 0.26 mm

#### Data collection

Bruker SMART APEXII CCD diffractometer
Absorption correction: multi-scan (*SADABS*; Bruker, 2009 ▶) *T*_min_ = 0.908, *T*_max_ = 0.937
14376 measured reflections
3600 independent reflections
3131 reflections with *I* > 2σ(*I*)
*R*_int_ = 0.025

#### Refinement

*R*[*F* ^2^ > 2σ(*F* ^2^)] = 0.038
*wR*(*F* ^2^) = 0.101
*S* = 1.02
3600 reflections
209 parameters
H-atom parameters constrained
Δρ_max_ = 0.45 e Å^−3^
Δρ_min_ = −0.35 e Å^−3^

### 

Data collection: *APEX2* (Bruker, 2009 ▶); cell refinement: *SAINT* (Bruker, 2009 ▶); data reduction: *SAINT*; program(s) used to solve structure: *SHELXS97* (Sheldrick, 2008 ▶); program(s) used to refine structure: *SHELXL97* (Sheldrick, 2008 ▶); molecular graphics: *ORTEP-3* (Farrugia, 1997 ▶) and *DIAMOND* (Brandenburg, 1998 ▶); software used to prepare material for publication: *SHELXL97*.

## Supplementary Material

Crystal structure: contains datablock(s) global, I. DOI: 10.1107/S1600536812044832/mw2094sup1.cif

Structure factors: contains datablock(s) I. DOI: 10.1107/S1600536812044832/mw2094Isup2.hkl

Supplementary material file. DOI: 10.1107/S1600536812044832/mw2094Isup3.cml

Additional supplementary materials:  crystallographic information; 3D view; checkCIF report

## Figures and Tables

**Table 1:** Hydrogen-bond geometry (Å, °) *Cg*1 is the centroid of the C2–C7 benzene ring.

*D*—H⋯*A*	*D*—H	H⋯*A*	*D*⋯*A*	*D*—H⋯*A*
C9—H9*A*⋯O4^i^	0.99	2.45	3.314 (2)	145
C9—H9*B*⋯O2^ii^	0.99	2.59	3.519 (3)	156
C16—H16*A*⋯O4^iii^	0.99	2.29	3.2689 (19)	169
C6—H6⋯F1^iv^	0.95	2.37	3.2532 (18)	154
C17—H17*B*⋯*Cg*1^v^	0.98	2.74	3.642 (3)	153

Symmetry codes: (i) 
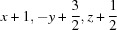
; (ii) 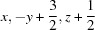; (iii) 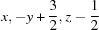; (iv) 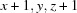; (v) 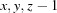.

**Fig. 1.**
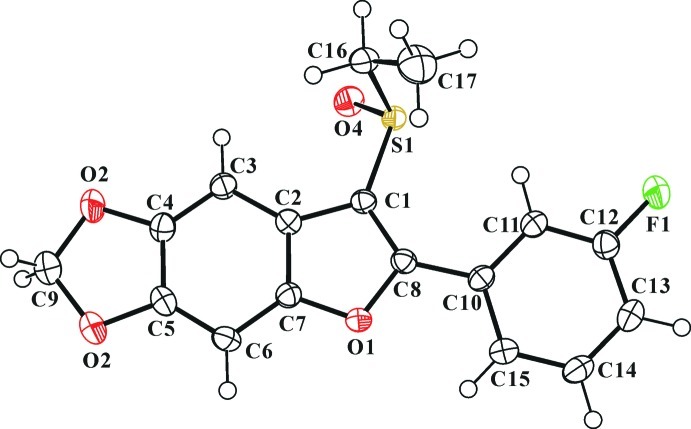
The molecular structure of the title compound with the atom numbering scheme. Displacement ellipsoids are drawn at the 50% probability level. H atoms are presented as small spheres of arbitrary radius.

**Fig. 2.**
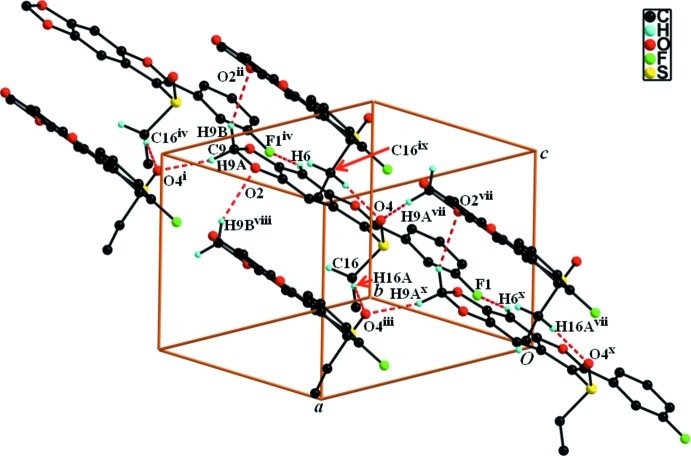
A view of the C—H···F and C—H···O interactions (dotted lines) in the crystal structure of the title compound. H atoms not participating in hydrogen-bonding were omitted for clarity. [Symmetry codes: (i) x + 1, - y + 3/2, z + 1/2; (ii) x, - y + 3/2, z + 1/2; (iii) x, - y + 3/2, z - 1/2; (iv) x + 1, y, z + 1 (vii) x - 1, - y + 3/2, z - 1/2; (viii) x, - y + 3/2, z + 1/2; (ix) x, - y + 3/2, z + 1/2; (x) x - 1, y, z - 1.]

**Fig. 3.**
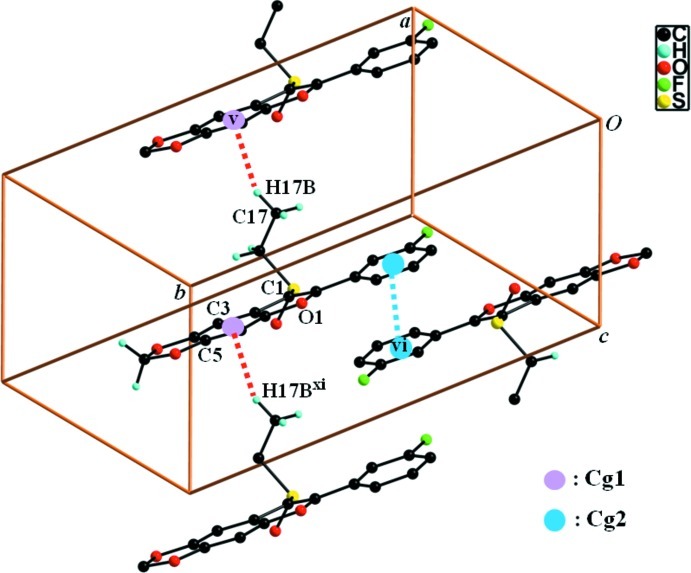
A view of the C—H···π and π–π interactions (dotted lines) in the crystal structure of the title compound. H atoms not participating in hydrogen-bonding were omitted for clarity. [Symmetry codes: (v) x, y, z - 1; (vi) - x, - y + 1, - z + 1; (xi) x, y, z + 1.]

## supplementary crystallographic information

### Comment

As a part of our ongoing study of 5,6-methylenedioxy-1-benzofuran derivatives containing [3-ethylsulfinyl-2-(4-fluorophenyl)] (Choi *et al.*, 2010) and [2-(4-fluorophenyl)-3-isopropylsulfinyl] (Seo *et al.*, 2011) substituents, we report herein the crystal structure of the title compound.

In the title molecule (Fig. 1), the benzofuran unit is essentially planar, with a mean deviation of 0.008 (1) Å from the least-squares plane defined by the nine constituent atoms. The dihedral angle between the 3-fluorophenyl ring and the mean plane of the benzofuran fragment is 6.14 (5)°. In the crystal structure (Figs. 2 & 3), molecules are connected by weak C—H···F, C—H···O and C—H···π interactions (Table 1, Cg1 is the centroid of the C2–C7 benzene ring). The crystal packing (Fig. 3) also exhibits slipped π–π interactions between the 3-fluorophenyl rings of neighbouring molecules, with a Cg2···Cg2^vi^ distance of 3.769 (2) Å and an interplanar distance of 3.372 (2) Å resulting in a slippage of 1.684 (2) Å (Cg2 is the centroid of the C10–C15 3-fluorophenyl ring).

### Experimental

3-Chloroperoxybenzoic acid (77%, 224 mg, 1.0 mmol) was added in small portions to a stirred solution of 3-ethylsulfanyl-2-(3-fluorophenyl)-5,6-methylenedioxy-1-benzofuran (284 mg, 0.9 mmol) in dichloromethane (40 mL) at 273 K. After being stirred at room temperature for 4h, the mixture was washed with saturated sodium bicarbonate solution and the organic layer was separated, dried over magnesium sulfate, filtered and concentrated at reduced pressure. The residue was purified by column chromatography (hexane–ethyl acetate, 1:1 v/v) to afford the title compound as a colorless solid [yield 73%, m.p. 408–409 K; *R*_f_ = 0.61 (hexane–ethyl acetate, 1:1 v/v)]. Single crystals suitable for X-ray diffraction were prepared by slow evaporation of a solution of the title compound in acetone at room temperature.

### Refinement

All H atoms were geometrically positioned and refined using a riding model, with C—H = 0.95 Å for aryl, 0.99 Å for methylene and 0.95 Å for methyl H atoms, respectively. *U*_iso_(H) = 1.2*U*_eq_(C) for aryl and methylene, and 1.5*U*_eq_(C) for methyl H atoms. The positions of methyl hydrogens were optimized rotationally.

### Crystal data

**Table:**

C_17_H_13_FO_4_S	*F*(000) = 688
*M**_r_* = 332.33	*D*_x_ = 1.518 Mg m^−^^3^
Monoclinic, *P*2_1_/*c*	Melting point: 408 K
Hall symbol: -P 2ybc	Mo *K*α radiation, λ = 0.71073 Å
*a* = 8.8516 (2) Å	Cell parameters from 6139 reflections
*b* = 21.8221 (4) Å	θ = 2.9–28.2°
*c* = 7.7228 (2) Å	µ = 0.25 mm^−^^1^
β = 102.949 (1)°	*T* = 173 K
*V* = 1453.80 (6) Å^3^	Block, colourless
*Z* = 4	0.39 × 0.29 × 0.26 mm

### Data collection

**Table:**

Bruker SMART APEXII CCD diffractometer	3600 independent reflections
Radiation source: rotating anode	3131 reflections with *I* > 2σ(*I*)
Graphite multilayer monochromator	*R*_int_ = 0.025
Detector resolution: 10.0 pixels mm^-1^	θ_max_ = 28.3°, θ_min_ = 1.9°
φ and ω scans	*h* = −11→11
Absorption correction: multi-scan (*SADABS*; Bruker, 2009)	*k* = −29→27
*T*_min_ = 0.908, *T*_max_ = 0.937	*l* = −10→10
14376 measured reflections	

### Refinement

**Table:**

Refinement on *F*^2^	Primary atom site location: structure-invariant direct methods
Least-squares matrix: full	Secondary atom site location: difference Fourier map
*R*[*F*^2^ > 2σ(*F*^2^)] = 0.038	Hydrogen site location: difference Fourier map
*wR*(*F*^2^) = 0.101	H-atom parameters constrained
*S* = 1.02	*w* = 1/[σ^2^(*F*_o_^2^) + (0.0488*P*)^2^ + 0.7371*P*] where *P* = (*F*_o_^2^ + 2*F*_c_^2^)/3
3600 reflections	(Δ/σ)_max_ = 0.001
209 parameters	Δρ_max_ = 0.45 e Å^−^^3^
0 restraints	Δρ_min_ = −0.35 e Å^−^^3^

### Special details

**Table:**

Geometry. All esds (except the esd in the dihedral angle between two l.s. planes) are estimated using the full covariance matrix. The cell esds are taken into account individually in the estimation of esds in distances, angles and torsion angles; correlations between esds in cell parameters are only used when they are defined by crystal symmetry. An approximate (isotropic) treatment of cell esds is used for estimating esds involving l.s. planes.
Refinement. Refinement of F^2^ against ALL reflections. The weighted R-factor wR and goodness of fit S are based on F^2^, conventional R-factors R are based on F, with F set to zero for negative F^2^. The threshold expression of F^2^ > 2sigma(F^2^) is used only for calculating R-factors(gt) etc. and is not relevant to the choice of reflections for refinement. R-factors based on F^2^ are statistically about twice as large as those based on F, and R- factors based on ALL data will be even larger.

### Fractional atomic coordinates and isotropic or equivalent isotropic displacement parameters (Å^2^)

**Table:**

	*x*	*y*	*z*	*U*_iso_*/*U*_eq_	
S1	0.20748 (4)	0.653717 (15)	0.40724 (5)	0.02313 (11)	
F1	−0.09845 (13)	0.46852 (5)	0.11643 (15)	0.0452 (3)	
O1	0.44774 (12)	0.52350 (4)	0.70767 (14)	0.0247 (2)	
O2	0.76433 (15)	0.73096 (6)	0.93233 (18)	0.0424 (3)	
O3	0.86160 (15)	0.64201 (6)	1.07899 (18)	0.0431 (3)	
O4	0.18119 (13)	0.70803 (5)	0.51487 (16)	0.0341 (3)	
C1	0.33826 (16)	0.60530 (6)	0.55117 (19)	0.0215 (3)	
C2	0.47112 (16)	0.62662 (6)	0.68258 (18)	0.0222 (3)	
C3	0.54231 (17)	0.68385 (7)	0.7276 (2)	0.0252 (3)	
H3	0.5037	0.7207	0.6683	0.030*	
C4	0.67160 (18)	0.68235 (7)	0.8635 (2)	0.0271 (3)	
C5	0.72968 (17)	0.62887 (7)	0.9530 (2)	0.0278 (3)	
C6	0.66316 (17)	0.57266 (7)	0.9140 (2)	0.0271 (3)	
H6	0.7020	0.5362	0.9755	0.033*	
C7	0.53247 (16)	0.57424 (6)	0.77457 (19)	0.0229 (3)	
C8	0.32896 (16)	0.54330 (6)	0.57115 (19)	0.0223 (3)	
C9	0.8859 (2)	0.70603 (8)	1.0666 (2)	0.0403 (4)	
H9A	0.9872	0.7137	1.0365	0.048*	
H9B	0.8866	0.7259	1.1821	0.048*	
C10	0.22658 (16)	0.49448 (6)	0.48447 (19)	0.0225 (3)	
C11	0.10942 (17)	0.50551 (7)	0.3331 (2)	0.0257 (3)	
H11	0.0948	0.5451	0.2812	0.031*	
C12	0.01626 (18)	0.45741 (7)	0.2619 (2)	0.0274 (3)	
C13	0.03117 (18)	0.39881 (7)	0.3285 (2)	0.0292 (3)	
H13	−0.0358	0.3668	0.2743	0.035*	
C14	0.14759 (19)	0.38828 (7)	0.4776 (2)	0.0314 (3)	
H14	0.1613	0.3483	0.5272	0.038*	
C15	0.24452 (18)	0.43523 (7)	0.5557 (2)	0.0280 (3)	
H15	0.3238	0.4272	0.6583	0.034*	
C16	0.33604 (19)	0.67847 (7)	0.2700 (2)	0.0306 (3)	
H16A	0.2947	0.7164	0.2061	0.037*	
H16B	0.4390	0.6880	0.3462	0.037*	
C17	0.3535 (2)	0.62964 (10)	0.1369 (3)	0.0450 (4)	
H17A	0.3961	0.5923	0.2000	0.068*	
H17B	0.4237	0.6443	0.0642	0.068*	
H17C	0.2519	0.6206	0.0600	0.068*	

### Atomic displacement parameters (Å^2^)

**Table:**

	*U*^11^	*U*^22^	*U*^33^	*U*^12^	*U*^13^	*U*^23^
S1	0.02202 (18)	0.01839 (17)	0.02712 (19)	0.00208 (12)	0.00156 (13)	0.00075 (13)
F1	0.0445 (6)	0.0362 (5)	0.0436 (6)	−0.0053 (4)	−0.0138 (5)	−0.0012 (4)
O1	0.0256 (5)	0.0197 (5)	0.0266 (5)	0.0015 (4)	0.0013 (4)	0.0023 (4)
O2	0.0397 (7)	0.0311 (6)	0.0465 (7)	−0.0092 (5)	−0.0113 (6)	−0.0031 (5)
O3	0.0365 (7)	0.0404 (7)	0.0419 (7)	−0.0063 (5)	−0.0133 (5)	0.0026 (5)
O4	0.0370 (6)	0.0226 (5)	0.0417 (7)	0.0079 (4)	0.0069 (5)	−0.0049 (5)
C1	0.0206 (6)	0.0208 (6)	0.0233 (6)	0.0025 (5)	0.0050 (5)	0.0011 (5)
C2	0.0216 (6)	0.0227 (7)	0.0226 (7)	0.0025 (5)	0.0056 (5)	−0.0009 (5)
C3	0.0261 (7)	0.0212 (7)	0.0278 (7)	0.0020 (5)	0.0046 (6)	−0.0011 (5)
C4	0.0264 (7)	0.0257 (7)	0.0288 (7)	−0.0023 (6)	0.0051 (6)	−0.0037 (6)
C5	0.0225 (7)	0.0342 (8)	0.0248 (7)	−0.0003 (6)	0.0011 (6)	−0.0010 (6)
C6	0.0262 (7)	0.0271 (7)	0.0264 (7)	0.0040 (6)	0.0021 (6)	0.0047 (6)
C7	0.0233 (7)	0.0211 (7)	0.0241 (7)	0.0002 (5)	0.0051 (5)	0.0000 (5)
C8	0.0219 (6)	0.0220 (7)	0.0225 (7)	0.0022 (5)	0.0041 (5)	0.0007 (5)
C9	0.0382 (9)	0.0403 (9)	0.0363 (9)	−0.0093 (8)	−0.0048 (7)	−0.0005 (7)
C10	0.0241 (7)	0.0196 (6)	0.0253 (7)	0.0001 (5)	0.0086 (5)	−0.0007 (5)
C11	0.0279 (7)	0.0212 (7)	0.0278 (7)	0.0001 (5)	0.0058 (6)	−0.0005 (5)
C12	0.0271 (7)	0.0274 (7)	0.0270 (7)	0.0003 (6)	0.0045 (6)	−0.0030 (6)
C13	0.0319 (8)	0.0242 (7)	0.0333 (8)	−0.0058 (6)	0.0111 (6)	−0.0059 (6)
C14	0.0391 (9)	0.0202 (7)	0.0360 (8)	−0.0007 (6)	0.0106 (7)	0.0017 (6)
C15	0.0308 (7)	0.0236 (7)	0.0289 (7)	0.0009 (6)	0.0050 (6)	0.0031 (6)
C16	0.0310 (8)	0.0296 (8)	0.0301 (8)	−0.0033 (6)	0.0046 (6)	0.0080 (6)
C17	0.0486 (11)	0.0550 (12)	0.0347 (9)	−0.0016 (9)	0.0162 (8)	−0.0018 (8)

### Geometric parameters (Å, º)

**Table:**

S1—O4	1.4954 (11)	C8—C10	1.4602 (19)
S1—C1	1.7651 (14)	C9—H9A	0.9900
S1—C16	1.8029 (16)	C9—H9B	0.9900
F1—C12	1.3568 (18)	C10—C11	1.399 (2)
O1—C7	1.3717 (17)	C10—C15	1.400 (2)
O1—C8	1.3809 (16)	C11—C12	1.372 (2)
O2—C4	1.3733 (18)	C11—H11	0.9500
O2—C9	1.424 (2)	C12—C13	1.373 (2)
O3—C5	1.3724 (18)	C13—C14	1.381 (2)
O3—C9	1.420 (2)	C13—H13	0.9500
C1—C8	1.366 (2)	C14—C15	1.385 (2)
C1—C2	1.4473 (19)	C14—H14	0.9500
C2—C7	1.3905 (19)	C15—H15	0.9500
C2—C3	1.407 (2)	C16—C17	1.513 (3)
C3—C4	1.369 (2)	C16—H16A	0.9900
C3—H3	0.9500	C16—H16B	0.9900
C4—C5	1.395 (2)	C17—H17A	0.9800
C5—C6	1.365 (2)	C17—H17B	0.9800
C6—C7	1.393 (2)	C17—H17C	0.9800
C6—H6	0.9500		
			
O4—S1—C1	106.63 (7)	O3—C9—H9B	109.9
O4—S1—C16	106.65 (7)	O2—C9—H9B	109.9
C1—S1—C16	97.95 (7)	H9A—C9—H9B	108.3
C7—O1—C8	107.07 (10)	C11—C10—C15	118.99 (13)
C4—O2—C9	105.97 (13)	C11—C10—C8	121.69 (13)
C5—O3—C9	105.99 (13)	C15—C10—C8	119.32 (13)
C8—C1—C2	107.41 (12)	C12—C11—C10	118.03 (14)
C8—C1—S1	128.05 (11)	C12—C11—H11	121.0
C2—C1—S1	124.35 (11)	C10—C11—H11	121.0
C7—C2—C3	120.10 (13)	F1—C12—C11	117.79 (14)
C7—C2—C1	104.88 (12)	F1—C12—C13	117.90 (13)
C3—C2—C1	135.01 (13)	C11—C12—C13	124.31 (14)
C4—C3—C2	114.80 (13)	C12—C13—C14	117.26 (14)
C4—C3—H3	122.6	C12—C13—H13	121.4
C2—C3—H3	122.6	C14—C13—H13	121.4
C3—C4—O2	127.07 (14)	C13—C14—C15	120.90 (14)
C3—C4—C5	123.50 (14)	C13—C14—H14	119.5
O2—C4—C5	109.43 (13)	C15—C14—H14	119.5
C6—C5—O3	126.94 (14)	C14—C15—C10	120.50 (14)
C6—C5—C4	123.35 (14)	C14—C15—H15	119.8
O3—C5—C4	109.70 (14)	C10—C15—H15	119.8
C5—C6—C7	113.02 (13)	C17—C16—S1	111.26 (12)
C5—C6—H6	123.5	C17—C16—H16A	109.4
C7—C6—H6	123.5	S1—C16—H16A	109.4
O1—C7—C2	110.68 (12)	C17—C16—H16B	109.4
O1—C7—C6	124.09 (12)	S1—C16—H16B	109.4
C2—C7—C6	125.22 (13)	H16A—C16—H16B	108.0
C1—C8—O1	109.95 (12)	C16—C17—H17A	109.5
C1—C8—C10	135.85 (13)	C16—C17—H17B	109.5
O1—C8—C10	114.20 (12)	H17A—C17—H17B	109.5
O3—C9—O2	108.90 (13)	C16—C17—H17C	109.5
O3—C9—H9A	109.9	H17A—C17—H17C	109.5
O2—C9—H9A	109.9	H17B—C17—H17C	109.5
			
O4—S1—C1—C8	134.07 (14)	C1—C2—C7—C6	179.62 (14)
C16—S1—C1—C8	−115.83 (15)	C5—C6—C7—O1	178.29 (14)
O4—S1—C1—C2	−40.34 (14)	C5—C6—C7—C2	−0.7 (2)
C16—S1—C1—C2	69.76 (13)	C2—C1—C8—O1	0.08 (16)
C8—C1—C2—C7	−0.34 (16)	S1—C1—C8—O1	−175.09 (10)
S1—C1—C2—C7	175.05 (11)	C2—C1—C8—C10	−179.64 (16)
C8—C1—C2—C3	178.82 (16)	S1—C1—C8—C10	5.2 (3)
S1—C1—C2—C3	−5.8 (2)	C7—O1—C8—C1	0.22 (16)
C7—C2—C3—C4	0.3 (2)	C7—O1—C8—C10	180.00 (12)
C1—C2—C3—C4	−178.78 (16)	C5—O3—C9—O2	0.8 (2)
C2—C3—C4—O2	178.99 (15)	C4—O2—C9—O3	−1.3 (2)
C2—C3—C4—C5	−0.4 (2)	C1—C8—C10—C11	5.9 (3)
C9—O2—C4—C3	−178.18 (17)	O1—C8—C10—C11	−173.83 (13)
C9—O2—C4—C5	1.29 (19)	C1—C8—C10—C15	−173.27 (17)
C9—O3—C5—C6	178.63 (17)	O1—C8—C10—C15	7.0 (2)
C9—O3—C5—C4	−0.03 (19)	C15—C10—C11—C12	0.5 (2)
C3—C4—C5—C6	0.0 (3)	C8—C10—C11—C12	−178.70 (14)
O2—C4—C5—C6	−179.54 (15)	C10—C11—C12—F1	179.18 (13)
C3—C4—C5—O3	178.68 (15)	C10—C11—C12—C13	−0.6 (2)
O2—C4—C5—O3	−0.81 (19)	F1—C12—C13—C14	−179.45 (14)
O3—C5—C6—C7	−177.91 (15)	C11—C12—C13—C14	0.3 (2)
C4—C5—C6—C7	0.6 (2)	C12—C13—C14—C15	0.1 (2)
C8—O1—C7—C2	−0.45 (16)	C13—C14—C15—C10	−0.2 (2)
C8—O1—C7—C6	−179.59 (14)	C11—C10—C15—C14	−0.1 (2)
C3—C2—C7—O1	−178.82 (13)	C8—C10—C15—C14	179.08 (14)
C1—C2—C7—O1	0.49 (16)	O4—S1—C16—C17	−172.19 (12)
C3—C2—C7—C6	0.3 (2)	C1—S1—C16—C17	77.73 (13)

### Hydrogen-bond geometry (Å, º)

Cg1 is the centroid of the C2–C7 benzene ring.

**Table:**

*D*—H···*A*	*D*—H	H···*A*	*D*···*A*	*D*—H···*A*
C9—H9*A*···O4^i^	0.99	2.45	3.314 (2)	145
C9—H9*B*···O2^ii^	0.99	2.59	3.519 (3)	156
C16—H16*A*···O4^iii^	0.99	2.29	3.2689 (19)	169
C6—H6···F1^iv^	0.95	2.37	3.2532 (18)	154
C17—H17*B*···*Cg*1^v^	0.98	2.74	3.642 (3)	153

Symmetry codes: (i) *x*+1, −*y*+3/2, *z*+1/2; (ii) *x*, −*y*+3/2, *z*+1/2; (iii) *x*, −*y*+3/2, *z*−1/2; (iv) *x*+1, *y*, *z*+1; (v) *x*, *y*, *z*−1.

## References

[bb1] Brandenburg, K. (1998). *DIAMOND* Crystal Impact GbR, Bonn, Germany.

[bb2] Bruker (2009). *APEX2*, *SADABS* and *SAINT* Bruker AXS Inc., Madison, Wisconsin, USA.

[bb3] Choi, H. D., Seo, P. J., Son, B. W. & Lee, U. (2010). *Acta Cryst.* E**66**, o1296. 10.1107/S160053681001617X PMC2979611 21579393

[bb4] Farrugia, L. J. (1997). *J. Appl. Cryst.* **30**, 565.

[bb5] Seo, P. J., Choi, H. D., Son, B. W. & Lee, U. (2011). *Acta Cryst.* E**67**, o3504. 10.1107/S1600536811051178 PMC3239126 22199974

[bb6] Sheldrick, G. M. (2008). *Acta Cryst.* A**64**, 112–122. 10.1107/S0108767307043930 18156677

